# Protein arginine methyltransferase 7 promotes breast cancer cell invasion through the induction of MMP9 expression

**DOI:** 10.18632/oncotarget.3072

**Published:** 2014-12-26

**Authors:** R. Mitchell Baldwin, Nasim Haghandish, Manijeh Daneshmand, Shahrier Amin, Geneviève Paris, Theresa J. Falls, John C. Bell, Shahidul Islam, Jocelyn Côté

**Affiliations:** ^1^ Department of Cellular and Molecular Medicine, Ottawa, Ontario, Canada; ^2^ Faculty of Medicine, University of Ottawa, Ottawa, Ontario, Canada; ^3^ Center for Innovative Cancer Therapeutics, Ottawa Hospital Research Institute, Ottawa, Ontario, Canada; ^4^ Department of Pathology and Laboratory Medicine, University of Ottawa, Ottawa, Ontario, Canada; ^5^ Department of Pathology, Ottawa Hospital, Ottawa, Ontario, Canada; ^6^ Department of Biochemistry, Microbiology and Immunology, University of Ottawa, Ottawa, Ontario, Canada

**Keywords:** Protein arginine methyltransferase, PRMT7, breast cancer, invasion, MMP9

## Abstract

Recent evidence points to the protein arginine methyltransferase (PRMT) family of enzymes playing critical roles in cancer. PRMT7 has been identified in several gene expression studies to be associated with increased metastasis and decreased survival in breast cancer patients. However, this has not been extensively studied. Here we report that PRMT7 expression is significantly upregulated in both primary breast tumour tissues and in breast cancer lymph node metastases. We have demonstrated that reducing PRMT7 levels in invasive breast cancer cells using RNA interference significantly decreased cell invasion *in vitro* and metastasis *in vivo*. Conversely, overexpression of PRMT7 in non-aggressive MCF7 cells enhanced their invasiveness. Furthermore, we show that PRMT7 induces the expression of matrix metalloproteinase 9 (MMP9), a well-known mediator of breast cancer metastasis. Importantly, we significantly rescued invasion of aggressive breast cancer cells depleted of PRMT7 by the exogenous expression of MMP9. Our results demonstrate that upregulation of PRMT7 in breast cancer may have a significant role in promoting cell invasion through the regulation of MMP9. This identifies PRMT7 as a novel and potentially significant biomarker and therapeutic target for breast cancer.

## INTRODUCTION

Breast cancer is one of the most common cancer types affecting women as it is expected that 1 in 9 women will develop breast cancer in their lifetime (Statistics Canada, 2013). Despite the advances in early detection methods and aggressive treatment strategies, breast cancer is still a leading cause of cancer related deaths. These deaths are mainly due to tumour recurrence [1]. These recurrent tumours are often more aggressive, possess an intrinsic resistance to therapy, and have a high metastatic potential, all of which contribute to a poor prognosis. Identifying biological events and molecular targets associated with these phenomena will lead to improved therapeutics and a better prognosis [2].

Arginine methylation is a common post-translational modification catalyzed by protein arginine methyltransferases (PRMTs). These enzymes catalyze the transfer of a methyl group from S-adenosyl methionine (AdoMet; a methyl donor) to a guanidine nitrogen atom of arginine residues. PRMTs regulate several cellular processes including signal transduction, protein interactions, DNA repair, protein subcellular localization and RNA processing [3, 4]. Currently, there have been eleven reported PRMT enzyme family members. In mammalian cells, there are eight well-characterized PRMTs that are classified into three main types based on the methylation reaction they catalyze. Type I (PRMT1, 2, 3, 4 (CARM1), 6, 8) and Type II (PRMT5) are capable of generating mono-methylarginines as well as asymmetric and symmetric dimethylarginines (ADMA and SDMA), respectively [3, 5, 6]. Type III (PRMT7) can only generate mono-methylarginine (MMA) which may represent a priming mark for the other enzyme types [7, 8], although this has yet to be demonstrated experimentally. Additionally, PRMT9 (4q31) has been shown to be structurally similar to PRMT7, however, its enzymatic activity has not been characterized [6, 9]. Finally, two F-box only family members, FBXO10 and FBXO11, show sequence similarities with the PRMT family but, have yet to be extensively studied [3, 10]. The list of arginine methylated protein substrates by PRMTs is constantly growing, and along with it the discovery of new functional roles and their involvement in the development and progression of cancer [11-16].

There have been a number of studies linking PRMTs to cancer [17-26]. For instance, PRMT1 is a component of an oncogenic transcriptional complex regulating MLL-mediated cell transformation [20]. PRMT1 methylates ERα in breast cancer cells promoting its extranuclear signaling and its interaction with PI-3 kinase and Src [27, 28]. PRMT1 has also been shown to regulate the DNA binding ability of key DNA repair proteins, MRE11 and 53BP1, and their function in DNA repair [29, 30]. We have previously shown that the alternatively spliced PRMT1 isoform, PRMT1v2, promotes breast cancer cell survival and invasion [31]. CARM1 influences hormone-dependent cell proliferation in prostate and breast cancer [19, 32]. PRMT5 expression is upregulated in mantle cell lymphoma and causes increased anchorage-independent growth [25, 33]. Additionally, PRMT5 methylates both p53 and E2F1, promoting a cell growth and survival advantage [34, 35]. Recently, PRMT6 has been linked to cancer through the transcriptional repression of TSP1, p21 and p53 [36-38]. These findings make PRMTs attractive targets for therapeutic intervention.

PRMT7 was first identified as a gene involved in regulating the cellular response to cytotoxic and chemotherapeutic agents [39]. It has been shown to be involved in the regulation of cellular resistance or sensitivity to cytotoxic agents depending on the drug or cell context [39-42]. PRMT7 also potentially influences male germline imprinted gene regulation through an interaction with CCCTC-binding factor-like protein (CTCFL) resulting in DNA methylation [43]. Interestingly, CTCFL has also been characterized as an oncogene and has been shown to be aberrantly expressed in several cancer types including breast cancer [44, 45]. Recent evidence has shown that PRMT7 inhibits neuronal cell differentiation through the repression of MLL4-dependent gene expression [46]. This may have an impact on the maintenance of a stem cell population, and has potential implications in cancer.

To date, no genetic alterations have been identified for the *PRMT7* gene, however it is located in a genomic region known to display high copy number abnormalities in breast cancer [47]. An assessment of 1200 breast tumour samples identified that the chromosomal region containing the *PRMT7* gene (16q22) is often associated with metastatic breast tumour samples and also associated with decreased patient survival. Furthermore, an assessment of PRMT7 expression using the unbiased genome-wide databases, ‘The Cancer Genome Atlas’, ‘Oncomine’ and the ‘ABREN’ revealed that it is dysregulated in breast cancer. Data from these consortiums suggest that a significant proportion of breast cancers display increased PRMT7 gene expression.

Here we have shown that PRMT7 protein expression is significantly increased in primary breast tumour tissues and breast cancer metastatic tissues. PRMT7 protein expression is also increased in highly invasive breast cancer cell lines. We have shown that specific knockdown of PRMT7 using RNA interference resulted in decreased breast cancer cell invasion *in vitro* and also metastasis *in vivo*. Alternatively, overexpression of PRMT7 in a non-aggressive breast cancer cell line caused them to become more invasive. This occurs, at least in part, through the induction of matrix metalloproteinase 9 (MMP9) expression, as exogenous expression of MMP9 in PRMT7-depleted cells enhanced their invasive capability. These results demonstrate that PRMT7 represents an attractive biomarker and therapeutic target for breast cancer.

## RESULTS

### PRMT7 is overexpressed in breast cancer tissue

**Figure 1:**
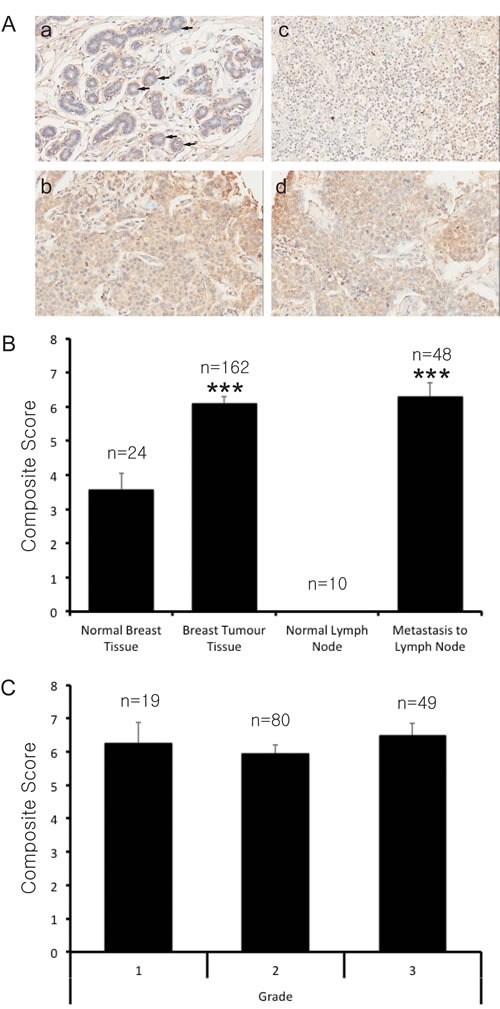
PRMT7 is overexpressed in breast cancer tissues High-density tissue microarrays were used to assess PRMT7 expression by immunohistochemistry in normal breast tissues, primary breast tumour tissues, normal lymph nodes and lymph nodes containing metastatic breast cancer. A, Representative images of normal breast tissue (a), primary breast tumour tissue (b), normal lymph node (c) and metastatic breast cancer (d) tissue samples are shown. Arrows highlight normal epithelial cells. Two pathologists independently scored the PRMT7 staining for each of the tissues examined. The mean composite (intensity × distribution) score for the normal breast tissues, primary breast tumour tissues, normal lymph nodes and lymph nodes containing metastatic disease are shown (B). An assessment of the composite scores for primary breast tumours with respect to grade was also examined (C). The number of samples for each of the tissue types is indicated (n). ***p < 0.0001.

It has been suggested from previous unbiased studies and through mining online expression databases that PRMT7 gene expression is dysregulated in breast cancer [47]. However, this has not been extensively examined experimentally. We assessed PRMT7 protein expression in breast cancer tissues by immunohistochemistry. We initially thoroughly validated a PRMT7 polyclonal antibody for use in this study and have shown that it specifically detects PRMT7 in Western blots of breast cancer cell lines with no other non-specific activity (Supplementary Figure 1A). Additionally, we observed no signal generated from our secondary antibody binding non-specifically in primary breast cancer tissues (Supplementary Figure 1B). To assess the expression of PRMT7 in breast cancer tissues, we used four commercially available high-density tissue microarrays (TMA). In total we evaluated the expression of PRMT7 in 244 tissue samples comprising of 24 normal breast tissues, 162 primary breast tumours, 48 lymph node metastases and 10 normal lymph nodes. Representative images of PRMT7 immunohistochemical staining in the tissues are shown in Figure 1A. An initial assessment of the PRMT7 staining in tissues showed that while it was present in the cytoplasm and nucleus of cells, a more intense staining was observed in the cytoplasm. Therefore, the cytoplasmic staining intensity was our focus for scoring. This localization of PRMT7 is consistent with what we have observed for endogenous PRMT7 by immunofluorescence in breast cancer cells (Supplementary Figure 1C) and also with previous observations [48].

Breast cancers mainly develop within the glandular cells of the breast and are characterized as epithelial-derived tumours. Therefore the staining intensities within epithelial cells of normal breast tissues and epithelial-derived breast tumour cells were evaluated. Of note, PRMT7 staining was examined in other cell types within normal breast tissues and we found that endothelial cells had a moderate staining level while scattered fibroblasts within the connective tissue were either negative or very weakly positive. Two pathologists scored PRMT7 staining intensity and distribution within the epithelial cells of the normal breast tissues and the epithelial-derived breast tumour cells to generate a composite score (intensity score multiplied by distribution score) for each of the tissue samples examined. The mean composite scores for each of the tissue groups are shown in Figure 1B. Our results show that the mean composite score of primary breast tumours were significantly higher than normal breast tissues, at 6.10 versus 3.58, respectively. Furthermore, the expression of PRMT7 protein was elevated in lymph node metastases originating from primary breast tumours (composite score of 6.29). The PRMT7 composite scores for breast tumour tissues and metastatic tissues were not significantly different. Normal lymph nodes were completely negative for PRMT7 expression and were void of epithelial cells. An assessment of the composite score distribution demonstrated that a significant proportion of breast cancer tissues (68%) and lymph node metastases (68%) had a strong expression (6+ composite score) of PRMT7 (Table 1). In contrast, the majority of normal breast tissues had a weak PRMT7 expression (42%; 0-2 composite score). No differences in PRMT7 expression were observed with increasing tumour grade when comparing mean composite scores (Figure 1C). However, a slightly higher proportion (76% versus 63%) of Grade III tumour samples exhibited high PRMT7 expression (composite score equal to or greater than 6) when compared to the Grade I/II tumour group, but this did not reach significance (Table 2). This evidence suggests that PRMT7 expression may increase in early tumour development and then remain elevated in metastatic disease.

**Table 1:** Summary of PRMT7 composite scoring distribution

Composite Score	Normal breast tissue (n = 24)	Primary breast tumour tissue (n = 162)	Lymph node metastasis (n = 48)
0-2 (low expression)	10 (42%)	16 (10%)	6 (13%)
3-5 (moderate expression)	7 (29 %)	36 (22%)	9 (19%)
6+ (high expression)	7 (29%)	110 (68%)	33 (68%)
P-values	0.0002^1^	0.000035^2^	0.0033^3^

NOTE: p-values calculated by χ^2^ test.

1 Comparing all three groups.

2 Comparing Normal breast tissue to Primary breast tumour tissue.

3 Comparing Normal breast tissue to Lymph node metastasis.

**Table 2:** Summary of PRMT7 composite scoring distribution with respect to breast tumour grade

Composite Score	Grade I/II (n = 99)	Grade III (n = 49)
< 6	37 (37%)	12 (24%)
≥ 6	62 (63%)	37 (76%)
P-value	0.11	

NOTE: p-value calculated by χ^2^ test.

### PRMT7 is overexpressed in highly invasive breast cancer cells

**Figure 2:**
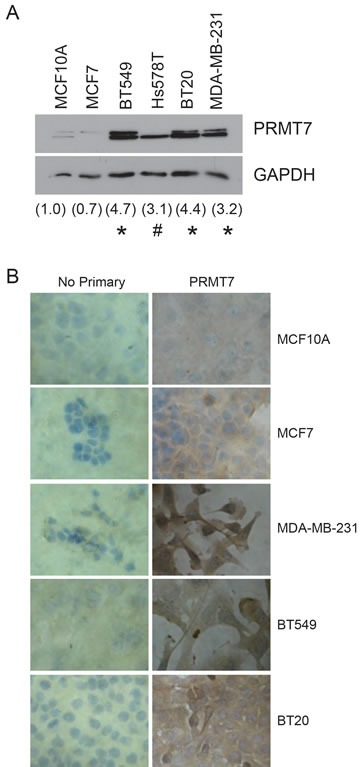
PRMT7 is overexpressed in breast cancer cell lines Western blot analysis for PRMT7 expression in established breast cancer cell lines (A). Densitometry of the band intensity (indicated below) were determined from 4 independent experiments and normalized to GAPDH and non-tumourigenic breast cells, MCF10A. *p < 0.05, #p = 0.13. Immunocytochemistry for PRMT7 expression was assessed in MCF10A, MCF7, MDA-MB-231, BT549 and BT20 cells (B). No primary antibody (No Primary) was used as a negative control.

**Figure 3:**
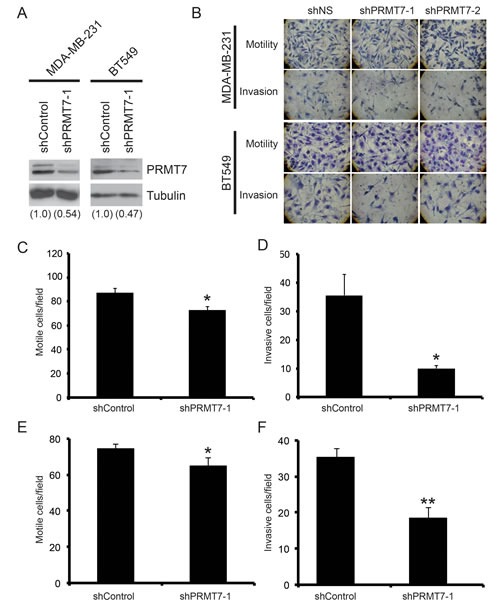
Knockdown of PRMT7 in invasive breast cancer cells inhibits their ability to invade PRMT7 was stably depleted in MDA-MB-231 and BT549 using lentiviral delivery of a PRMT7-targeting shRNA. A non-targeting shRNA was used as a control (shControl). Western analysis for PRMT7 in stably depleted MDA-MB-231 and BT549 cells (A). Tubulin serves as a loading control. Densitometry of the band intensities are indicated below in parentheses. MDA-MB-231 and BT549 cells depleted of PRMT7 were assessed for effects on motility and invasion. Cells were plated at equal numbers into Transwell chambers and incubated for 24 h. Motility was analyzed using Transwell chambers without a Matrigel (Motility). Invasion was analyzed using Transwell chambers containing a Matrigel layer (Invasion). Representative images of cells that have passed through the Transwell chamber ± Matrigel at 40X magnification (B). MDA-MB-231 and BT549 cell numbers that passed through the Transwell chambers in the absence of Matrigel (C and E, respectively: motile cells/field) or in the presence of Matrigel (D, MDA-MB-231 and F, BT549: invasive cells/field) were determined. Data represents the mean ± standard error of four independent experiments for MDA-MB-231 and three independent experiments for BT549 (*p < 0.05, **p < 0.01 comparing to control).

We next examined the expression of PRMT7 in established human breast cancer cell lines in order to corroborate our observations in human breast tumour tissues and validate their use as an experimental model. To quantitate the expression levels of PRMT7 protein within these cell lines Western blotting followed by band density analysis was performed (Figure 2A). PRMT7 protein expression was significantly higher (~3 to 5-fold) in highly invasive breast cancer cells compared to MCF10A and MCF7 cells. This evidence supports our observation that PRMT7 is overexpressed in breast cancer, and more specifically invasive cancer cells. Interestingly, two closely migrating bands are detected for PRMT7 in several breast cancer cell lines by Western blot. Both of these bands are reduced with PRMT7-specific shRNA (see below, Figure 3A), suggesting that they both represent PRMT7 species. Additional studies are required to determine the precise nature and origin of these PRMT7 species and their significance in terms of overall PRMT7 activity in cells. The presence of alternative isoforms is a common feature among the PRMT family of enzymes, as alternatively spliced isoforms have been identified for PRMT1, 2, and 4 [17, 41, 49-54]. Additionally, using the spontaneously immortalized non-tumourigenic breast epithelial cell line, MCF10A, as a control, the expression of PRMT7 was assessed by immunocytochemistry under similar conditions to the experiments performed on the human tissue samples. Consistent with our Western blot analysis, the weakly invasive breast cancer cell line, MCF7, had a staining intensity similar to MCF10A cells. In contrast, the intensity of staining in invasive breast cancer cell lines (MDA-MB-231, BT549 and BT20) [55] was much higher compared to the aforementioned cell lines (Figure 2B).

### Reducing PRMT7 expression levels inhibits breast cancer cell invasion

The ability of cancer cells to metastasize comprises two main characteristics: cell migration and their capacity to penetrate through a physical barrier such as the extracellular matrix of surrounding tissues. Due to the fact that PRMT7 is overexpressed in invasive breast cancer cells, we wanted to assess its importance in promoting cell invasiveness. To examine the role of PRMT7 in cancer cell motility and invasion we used the highly invasive breast cancer cell lines, MDA-MB-231 and BT549. PRMT7 was specifically and effectively knocked down from cells using lentiviral delivered short hairpin RNA (shPRMT7-1) and stable cell populations were established following selection. This allowed for a stable reduction of PRMT7 protein expression levels in both MDA-MB-231 cells and BT549 cells (Figure 3A). Control cells stably expressing a non-targeting shRNA sequence (shControl) were also established. A similar knockdown was also observed using a second unrelated PRMT7 shRNA targeted sequence (shPRMT7-2, Supplementary Figure 2A and B). To assess the effect of PRMT7 on cell motility and invasion, we used Transwell chamber assays. For this, control cells or PRMT7-depleted cells were counted and the same numbers were plated into Transwell chambers. Cells that crossed the chamber membrane were counted after a 24 h incubation. PRMT7 knockdown had a limited effect on cell motility causing either a slight reduction or no effect in invasive breast cancer cells (Figure 3C and E, Supplementary Figure 2C and E). To determine the effects of PRMT7 on invasion (cell motility combined with their ability to penetrate through physical barriers), we assessed the ability of invasive breast cancer cells with PRMT7 knockdown to migrate through Transwell chamber membranes coated with Matrigel. PRMT7 knockdown caused a significant decrease in the number of cells that invaded through Matrigel-coated membranes compared to controls. In MDA-MB-231 and BT549 cells, PRMT7 protein levels decreased 72% and 48% respectively using shPRMT7-1 (Figure 3D and F). Similarly, knockdown of PRMT7 with shPRMT7-2 resulted in a 56% and 40% decrease in invasion in MDA-MB-231 and BT549 cells, respectively (Supplementary Figure 2D and F). Additionally, we also isolated clonal populations of MDA-MB-231 cells stably expressing shPRMT7-1 and shPRMT7-2 (cl. 2 and cl. 3, respectively) and observed similar results (Supplementary Figure 3). While we do observe an effect of PRMT7 knockdown on cell growth (Supplementary Figure 4A, B and C), it is negligible at the time-point used for these experiments.

### Overexpression of PRMT7 enhances invasion of weakly invasive breast cancer cells

**Figure 4:**
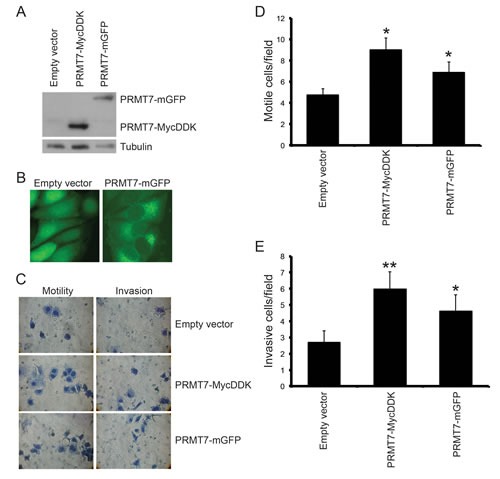
Overexpression of PRMT7 in non-invasive breast cancer cells promotes invasion PRMT7 was stably overexpressed in MCF7 cells by lentiviral transduction. An empty vector expressing mGFP was used as a control. Total protein lysates from MCF7 cells stably expressing empty vector, PRMT7-MycDDK or PRMT7-GFP were analyzed by Western blotting for PRMT7 expression (A). Tubulin serves as a loading control. B, Fluorescence images (40X) of MCF7 cells stably expressing an empty vector or PRMT7-GFP. MCF7 cells stably expressing PRMT7-MycDDK or PRMT7-GFP were analyzed for motility and invasion using Transwell chambers as previously described. However, cells that crossed the chamber membrane were counted following a 72 h incubation. Representative images of cells that have passed through the Transwell chamber ± Matrigel at 40X magnification (C). Cells that passed through the chamber membranes without a Matrigel layer (D: motile cells/field) or containing a Matrigel layer (E: invasive cells/field) were counted. Data represents the mean ± standard error of eleven independent experiments for PRMT7-MycDDK and nine independent experiments for PRMT7-GFP (*p < 0.05, **p < 0.01 comparing to Empty vector control).

To determine if PRMT7 could promote breast cancer cell invasion, we overexpressed PRMT7 in MCF7 cells. MCF7 cells are a well differentiated, “luminal epithelial-like” breast cancer cell line and characterized as weakly invasive breast cancer cells [55]. Importantly, as shown above, these cells expressed low PRMT7 protein levels compared to the highly invasive cell lines. To determine if PRMT7 is capable of enhancing the invasive capacity of these cells, we generated cell lines that stably expressed either a MycDDK or mGFP-tagged PRMT7 by lentiviral infection (Figure 4A). Cells expressing mGFP alone were generated as controls (Empty vector). Furthermore, the subcellular localization of our PRMT7-GFP fusion protein is consistent with what we observed for endogenous PRMT7 in breast cancer cells (Figure 4B). Once established, we examined their migration and invasion as previously described using Transwell chambers. As predicted from the literature, MCF7 cells displayed a low propensity to migrate through Transwell chamber membranes, even in the absence of Matrigel (Figure 4C). However, stable overexpression of PRMT7-MycDDK or PRMT7-mGFP enhanced by 1.9-fold and 1.5-fold, respectively, the ability of MCF7 cells to migrate, as compared to control cells (Figure 4C, D). Furthermore, when we assessed invasion through Transwell chambers containing Matrigel, PRMT7 overexpression significantly increased the number of cells that were able to invade (2.2-fold for PRMT7-MycDDK and 1.7-fold for PRMT7-mGFP compared to control; Figure 4C, E). We also observed that PRMT7 overexpression did not significantly affect the proliferation of MCF7 cells, therefore this has no impact on the increase in invasion observed (Supplementary Figure 4D). Altogether, these results are highly suggestive that PRMT7 plays a significant role in promoting breast cancer cell invasion, a necessary characteristic leading to metastasis.

### Targeting PRMT7 reduces breast cancer cell invasion *in vivo*

**Figure 5:**
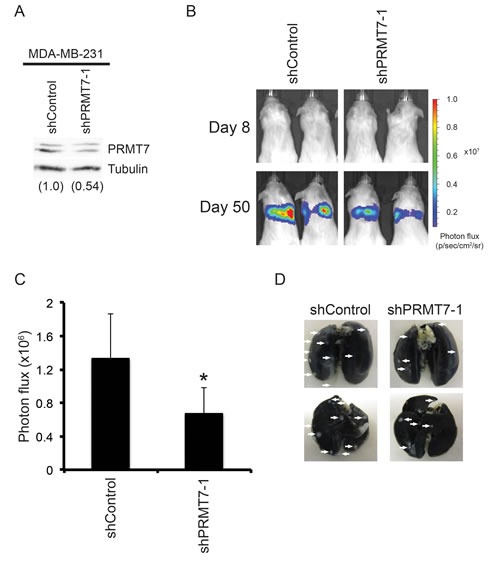
Knockdown of PRMT7 reduces breast cancer cell metastasis *in vivo* MDA-MB-231 cells stably expressing Luciferase and a control non-targeting shRNA or a PRMT7-targeting shRNA were injected into the tail vein of six week old female NOD.CB17-Prkdc^scid^/J SCID mice. Four mice were injected for each group. Western analysis for PRMT7 in stably depleted MDA-MB-231 into which the luciferase gene was introduced for ***in vivo*** imaging (A). Tubulin served as a loading control. Densitometry of the band intensities are indicated below in parentheses. Mice were injected intravenously and imaged using IVIS at day 8 and 50 post injection. Representative images of the bioluminescence (photon flux: p/s/cm^2^/sr) at day 8 and 50 are shown (B). Bioluminescence was quantitated for each mouse by measuring the photon flux (C). Data represents the mean ± standard error for each group (n = 4 mice/group, *p = 0.05). Representative images of whole lungs (anterior: upper image, posterior: lower image) stained with India ink, verifying the presence of cancer cell nodules upon the surface of the lungs (D).

To determine if PRMT7 knockdown affects breast cancer cell invasion *in vivo*, we performed an experimental metastasis study. In this model, human cancer cells are injected intravenously into the tail vein of immune compromised mice. The cells' ability to migrate through the blood stream and invade the lungs is then determined. MDA-MB-231 cells with stable PRMT7 knockdown (Figure 5A) were infected with a lentivirus containing the luciferase cDNA in order to introduce constitutive luciferase expression and allowing for visualization of these cells *in vivo* by IVIS. Cells expressing a non-targeting shRNA (shControl) and luciferase were used as controls for this study. Control or PRMT7 knockdown cells (500 000 cells) were injected into the tail vein of NOD.CB17-Prkdc^scid^/NrcCrl SCID mice that were randomly sorted into two groups of four mice (n = 4/group). On day 8 post injection, *in vivo* imaging was performed using IVIS (*In vivo* imaging system) to determine an initial bioluminescence signal. No signal was observed in either group at this time-point (Figure 5B). The extent of lung metastasis was evaluated 50 days post injection and, as expected, the bioluminescent signal was localized to the lung area. Importantly, mice injected with PRMT7-depleted cells showed a lower bioluminescent signal (photon flux: p/s/cm^2^/sr) compared to those injected with control shRNA expressing cells (Figure 5B). As a non-biased approached to measure the metastatic tumour burden within the lungs, the bioluminescent photon flux for each mouse was quantitated and the mean photon flux for each group was determined. This assessment showed that the group injected with PRMT7 knockdown cells had a significantly lower photon flux compared to the control cells (Figure 5C), thus indicating a reduction in the metastatic potential of cells *in vivo* with reduced PRMT7 levels. Lungs from these mice were dissected and stained to verify the presence of breast cancer cell nodules on the surface (Figure 5D). This data shows that PRMT7 has a role in promoting breast cancer cell metastasis *in vivo*.

### PRMT7 regulates the expression of matrix metalloproteinase 9, MMP9

**Figure 6:**
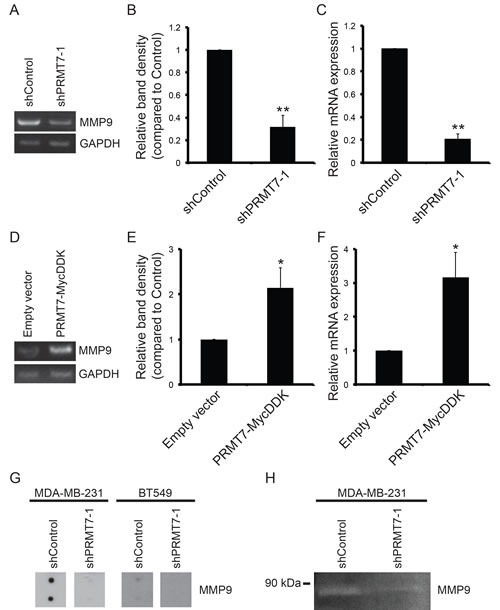
PRMT7 promotes MMP9 expression Total RNA was isolated from MDA-MB-231 cells stably depleted of PRMT7 and assessed for mRNA levels by RT-PCR and quantitative RT-PCR. Representative PCR (A) and band density quantitation of MMP9 mRNA levels (B) are shown. GAPDH served as a loading control. Data represents the mean ± standard error of six independent experiments (**p < 0.01). Quantitative RT-PCR analysis of MMP9 expression in PRMT7-depleted MDA-MB-231 cells (C). Data represents the mean ± standard error of five independent experiments (**p < 0.01). Total RNA was isolated from MCF7 cells stably expressing PRMT7-MycDDK and assessed for MMP9 mRNA levels by PCR analysis. Representative PCR (D) and band density quantitation of MMP9 mRNA levels (E) are shown. GAPDH served as a loading control. Data represents the mean ± standard error of four independent experiments (*p < 0.05). Quantitative RT-PCR analysis of MMP9 expression in MCF7 cells expressing PRMT7-MycDDK compared to empty vector control expressing cells (F). Data represents the mean ± standard error of four independent experiments (*p < 0.05). Secreted MMP9 protein levels were examined in conditioned media using an MMP antibody array. A reduction in secreted MMP9 protein levels was observed in both MDA-MB-231 and BT549 cells with PRMT7 knockdown (G). Gelatin zymography was used to determine the MMP9 enzymatic activity in conditioned media collected from MDA-MB-231 cells expressing a control shRNA (non-targeting) or PRMT7-targeted shRNA. PRMT7 depletion resulted in a reduction in MMP9 enzymatic activity (H).

Matrix metalloproteinases play a crucial role in cancer cell invasion [56]. These secreted proteins are responsible for the degradation of extracellular matrix proteins which allow cancer cells to invade local tissues, intravasate and extravasate blood vessels and lymphatic vessels, and form metastatic tumours at distant sites. MMP9 has been identified as a predictive marker of breast cancer cell invasion [57]. Therefore, we assessed the expression of MMP9 in invasive breast cancer cells depleted of PRMT7. In PRMT7-depleted MDA-MB-231 cells, a significant reduction in MMP9 mRNA was observed using both semi-quantitative and quantitative RT-PCR analysis, 68% and 79% decrease, respectively (Figure 6A, B, C). Decreased MMP9 mRNA expression was also observed in cells expressing shPRMT7-2 (Supplementary Figure 2G). Alternatively, in MCF7 cells stably overexpressing PRMT7, we observed a 2.1-fold increase in MMP9 mRNA expression by semi-quantitative RT-PCR (Figure 6D, E). Increased MMP9 mRNA expression in MCF7 PRMT7-MycDDK expressing cells was also confirmed by quantitative RT-PCR (3.2-fold increase, Figure 6F). MMP9 is secreted as an active protein that is capable of degrading collagen type IV [56, 58, 59]. To determine if this reduction in MMP9 mRNA expression also resulted in a decrease in MMP9 protein secretion, we used a membrane array containing MMP9 antibodies to detect protein levels in conditioned media. Conditioned media was collected from control (shControl) and PRMT7-knockdown MDA-MB-231 and BT549 cells and assessed for MMP9 protein levels by incubation with an MMP membrane array. Consistent with the decreased mRNA expression, we also observed a decrease in secreted MMP9 protein levels in both MDA-MB-231 and BT549 cells with PRMT7 knockdown (Figure 6G). Furthermore, consistent with a reduction in MMP9 expression and secretion, a decrease in MMP9 enzymatic activity was also observed using Gelatin zymography (Figure 6H), demonstrating the functional consequences that result from reduced expression. While the MMP arrays are able to detect other MMPs, we focused on MMP9 due to the significant impact it has on breast cancer cell invasion and metastasis. This evidence reveals that PRMT7 is capable of inducing MMP9 expression, representing a mechanism through which PRMT7 can promote breast cancer cell invasion.

### Exogenous re-expression of MMP9 in PRMT7-depleted invasive breast cancer cells rescues their invasive potential

**Figure 7:**
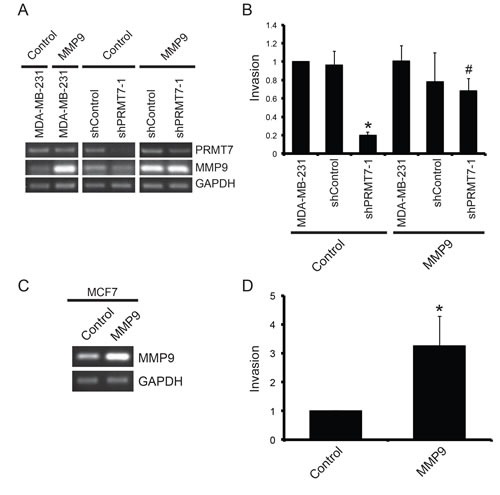
Overexpression of MMP9 rescues the loss of invasion resulting from PRMT7 depletion MDA-MB-231 cells stably depleted of PRMT7 using shRNA were transiently transfected with an empty vector (Control) or a vector containing MMP9 cDNA. Parental MDA-MB-231 (MDA-MB-231) and cells expressing a non-targeting shRNA cells were used as controls. Total RNA was collected 24 h post transfection and used to generate cDNA to assess PRMT7 and MMP9 expression levels (A). GAPDH served as a loading control. To examine the effect of MMP9 overexpression on cell invasion, MDA-MB-231 cells (MDA-MB-231), cells stably expressing a control shRNA (shControl) and MDA-MB-231 cells stably depleted of PRMT7 were transfected for 24 h as described above. Twenty-four hours post transfection, cells were re-plated at equal numbers into Transwell chambers containing a Matrigel layer and incubated for an additional 24 h. Cells that passed through the chambers were counted (B). Data represents the mean ± standard error of three independent experiments (*p < 0.05 compared to MDA-MB-231 cells stably expressing a control shRNA, #p < 0.05 compared to MDA-MB-231 cells stably depleted of PRMT7 transfected with an empty vector). MCF7 cells were transiently transfected with an empty vector (Control) or a vector containing MMP9 cDNA. Total RNA was collected 24 h post transfection to assess expression. PCR analysis of cDNA generated from total RNA using MMP9 primers shows expression levels (C). GAPDH serves as a loading control. To examine the effect of MMP9 overexpression on cell invasion, MCF7 cells were transfected for 24 h as described above and then replated at equal numbers into Transwell chambers containing a Matrigel layer and incubated for an additional 72 h. Cells that passed through the chambers were counted (D). Data represents the mean ± standard error of five independent experiments (*p < 0.05).

Lastly, to show that the observed effects were indeed the result of a change in MMP9 expression, we performed a rescue experiment in which we overexpressed MMP9 in PRMT7-knockdown cell lines. We rationalized that increasing MMP9 expression in cells that have reduced MMP9 levels (due to PRMT7 knockdown) should restore their invasive capability. MMP9 was exogenously expressed in breast cancer cells by transient transfection and assessed by PCR analysis of mRNA (Figure 7A). Following 24 h of transfection, cells were re-plated at equal numbers into Matrigel-coated Transwell chambers and incubated for an additional 24 h. As predicted, increased expression of MMP9 in PRMT7-depleted cells resulted in a significant rescue in the loss of invasion observed with PRMT7 depletion (Figure 7B). This demonstrates the importance of MMP9 in the invasive capacity of these cells. It is well established in the literature that an increase in MMP9 expression levels is associated with increased cancer cell invasion [56, 60-66]. Due to the fact that we observed an increase in MMP9 mRNA expression with PRMT7 overexpression in MCF7 cells which have low endogenous PRMT7 expression (Figure 6D, E, F), we wanted to know whether altering MMP9 expression alone is sufficient to affect their invasion. Not surprisingly, MMP9 expression caused a significant enhancement of MCF7 cell invasion (Figure 7C and D), demonstrating the direct role MMP9 expression has on breast cancer cell invasion. Taken together, these experiments demonstrate that PRMT7-regulated expression of MMP9 influences the invasive capabilities of breast cancer cells.

## DISCUSSION

In this study we have shown that PRMT7 plays a functional role in promoting breast cancer cell invasion. Our assessment of PRMT7 expression in breast tumour tissues has demonstrated that PRMT7 protein expression is upregulated in primary breast tumour tissues and breast cancer metastases. Consistent with this observation, established breast cancer cell lines, that are classified as highly invasive, poorly differentiated ‘mesenchymal-like’ cell lines, expressed significantly higher PRMT7 protein levels. Moreover, depletion of PRMT7 in highly invasive breast cancer cells using RNA interference decreased their invasiveness and ability to metastasize, *in vitro* and *in vivo* respectively. Alternatively, ectopic overexpression of PRMT7 in weakly invasive breast cancer cells enhanced their ability to invade. We identified a novel regulatory pathway in which PRMT7 induces the expression of matrix metalloproteinase 9 and showed that increased MMP9 expression significantly recues the loss of invasion observed with PRMT7 depletion. Overall, we have clearly established PRMT7 as a key mediator of breast cancer cell invasion and metastasis. In support of, and complementary to our findings, are the results described in a concurrent study published by Yao *et al.* showing a role for PRMT7 in promoting epithelial-to-mesenchymal transition (EMT) and breast cancer cell metastasis [67].

PRMT7 was initially characterized as possessing type II methyltransferase activity, capable of generating both mono-methylation [53] and symmetric di-methylation [54] of arginine residues of proteins *in vitro*. The precise product that it is capable of producing has often been debated however, two recent studies have now provided convincing evidence indicating that PRMT7 is exclusively a Type III enzyme, capable of only mono-methylating protein substrates at least *in vitro* [7, 8]. Furthermore, a consensus methylation site has also been recently identified and consists of an R-X-R motif [8]. There is currently limited knowledge describing the function(s) and substrate specificity of PRMT7; however, in light of this new data on the mono-methylating activity of PRMT7, its substrate repertoire is expected to increase more rapidly. This will provide more insight not only into its precise function in biological processes, but also a greater understanding into its potential role(s) in diseases, such as cancer.

Our rigorous immunohistochemical study of PRMT7 protein expression is the first to demonstrate that PRMT7 protein expression is significantly increased in primary breast tumours and lymph node metastases compared to normal breast tissues. PRMT7 expression was mainly localized to the cytoplasm of normal breast epithelial cells and breast tumour cells. This is consistent with the predominantly cytoplasmic localization of PRMT7 that has been previously established in various transformed cells [48]. We have shown that a significantly higher number of primary breast tumours and lymph node metastatic disease cases display high expression of PRMT7. Our results have also shown that a slightly higher proportion (76% versus 63%) of Grade III tumour samples exhibited high PRMT7 expression when compared to the Grade I/II tumour group, although this trend did not reach statistical significance with our sample size. Yao *et al.* have demonstrated that high PRMT7 expression levels in breast tumours correlates with clinicopathological features of aggressive breast tumours, including ER negative status and basal-like pathology [67]. Taken together, this expression data suggest that PRMT7 levels are increased at an early stage in breast tumour development where it plays a role in promoting EMT. Furthermore, elevated PRMT7 expression is maintained within the tumours potentially contributing to progression to metastatic disease. Additionally, PRMT7 may also play a significant role in other late hallmarks of cancer, such as chemoresistance (see below).

Consistent with these tissue expression analyses, we have shown that PRMT7 expression is significantly increased in a subset of established breast cancer cell lines compared to non-tumourigenic normal breast epithelial cells (MCF10A). Increased expression was observed in cell lines classified as more invasive with a ‘mesenchymal-like’ morphology (BT549, Hs578T, BT20 and MDA-MB-231). Whereas weakly invasive, ‘luminal epithelial-like’ cells, MCF7, expressed PRMT7 protein levels similar to MCF10A cells. Interestingly, each of the invasive cell lines examined is characterized genetically as triple negative (ER-, PR-, HER2-), whereas MCF7 cells are hormone receptor positive (ER+, PR+, HER2-) [68]. Thus further supporting the association of increased PRMT7 expression in aggressiveness of breast cancer cells.

Our examination of PRMT7 protein expression in breast cancer cells has also potentially uncovered an additional unique feature of PRMT7, the potential existence of alternative species. The presence of alternative isoforms is not uncommon among the PRMTs, as alternatively spliced isoforms have been identified for PRMT1, PRMT2 and CARM1 [17, 49-52]. These isoforms potentially have distinct roles in cancer cells [69]. Two closely migrating bands on a Western blot were often detected by PRMT7 antibodies. Intriguingly, we observed differential expression of these PRMT7 species. MCF7 cells only expressed the higher molecular weight form and Hs578T exclusively expressed the lower molecular weight form. The significance of this differential expression is not currently known. We believe that both these bands represent PRMT7 species due to the fact that PRMT7-targeted shRNAs are capable of knocking down both of their expression. Originally, in mammalian cells, it was found that the PRMT7 gene encoded two proteins, p77 (78kDa) and p82 (82kDa), that were highly homologous, and later designated PRMT7α and β, respectively [39, 41]. Several theories exist as to the origin of these two proteins. Firstly, in Chinese hamster cells, it is thought that these two isoforms are generated by the use of distinct 5′ translation initiation codons within the primary transcript, with the PRMT7β isoform containing an additional 37 amino acids at the N-terminus. In human tissues, only a single PRMT7 transcript was detected (~3.6 kb) and one protein, at 78kDa, was detected in two human cancer cell lines (HeLa and HuH7). This transcript was shown to share the greatest homology to the PRMT7α isoform [41, 53, 54]. However, the limited subset of cell lines used to conclude this cannot completely rule out the existence of the PRMT7β isoform expression in other human cell types. A more comprehensive examination of the expression of these isoforms in various cell types is required. Secondly, a survey within both NCBI and *Ensembl* databases predicts the existence of at least 2 alternatively spliced PRMT7 isoforms that can be produced from the human *PRMT7* gene. These two isoforms have the same N- and C-terminal regions however one has an in frame deletion of exon 5. Importantly, this may affect methyltransferase activity because it removes the post I domain. Finally, we cannot rule out the possibility that the higher molecular weight band is the result of one or more posttranslational modifications. Further studies into the precise nature and origin of these species are required in order to determine their specific functional roles in cells.

The association of PRMT7 expression with primary breast tumours and metastatic breast cancer led us to investigate whether PRMT7 directly affects breast cancer cell invasion. Metastasis of the primary tumour to distant sites is the major cause of mortality amongst breast cancer patients [70]. Here, we have shown that PRMT7 plays a direct role in promoting human breast cancer cell invasion. Depletion of PRMT7 in highly invasive breast cancer cells, MDA-MB-231 and BT549, caused a significant reduction in the ability of these cells to invade through an extracellular matrix-like barrier *in vitro*. Overexpression of PRMT7 in MCF7 cells, a weakly invasive breast cancer cell line, is sufficient to enhance their invasive capability. Furthermore, we showed that PRMT7 knockdown in MDA-MB-231 cells significantly reduced their ability to metastasize *in vivo*, demonstrating that targeting PRMT7 may be beneficial to inhibit this process. These results are consistent with those of Yao et al. [67], who have demonstrated that PRMT7 overexpression in MCF10A cells induced EMT and invasion *in vitro*, as well as metastasis *in vivo*. PRMT7-induced EMT was shown to involve the regulation of E-cadherin expression [67]. PRMT7 appears to be a component of a protein complex that interacts with the promoter of E-cadherin leading to the symmetric dimethylation of histone H4R3 [67]. This methyl mark resulted in the repression of E-cadherin expression. The recent classification of PRMT7 as a Type III arginine methyltransferase suggests that it may work in concert with other PRMT enzymes by catalyzing the initial priming mono-methyl arginine mark on protein substrates that can then be subsequently methylated by Type I or Type II PRMTs to produce an asymmetrically or symmetrically dimethylated arginine residues, respectively. However, more studies are necessary to experimentally establish this role for PRMT7-mediated mono-methylation.

A cancer cell's capacity to invade and metastasize is dependent on its ability to manipulate the tumour microenvironment in which it resides [71]. Key molecular mediators of tumour cells that promote cellular invasion are the matrix metalloproteinase family of zinc-dependent endopeptidases [56]. Matrix metalloproteinase 9 (MMP9, gelatinase B) production and secretion by tumour cells is a critical element involved in promoting metastasis [65]. MMP9 has been the focus of many studies due to the fact that it degrades Type IV collagen [58]. Type IV collagen is an abundant component of the basement membrane of epithelial cell layers. In breast cancer, MMP9 status is an important indicator of breast cancer prognosis [72]. Positive MMP9 expression is significantly associated with higher grade breast tumours (grades II and III) [56, 73]. Elevated levels of MMP9 expression is also associated with shortened relapse-free survival in breast cancer patients and a high rate of distant metastases [57]. Direct targeting of MMP9 expression in invasive breast cancer cells was shown to significantly inhibit cell invasion and metastasis *in vitro* and *in vivo* [65, 74]. The activity of MMP9 has also been shown to play a crucial role in breast cancer metastasis to the bone [75]. In an *in vivo* experimental model of bone metastasis, the presence of active MMP9 at the tumour-bone interface was important for bone destruction, a necessary process for tumour formation. Our assessment of MMP9 expression in invasive breast cancer cells showed that PRMT7 knockdown caused a significant decrease in MMP9 mRNA levels, secreted MMP9 protein amounts and decreased enzymatic activity. In turn, overexpression of PRMT7 in MCF7 cells resulted in a significant increase in MMP9 expression. A comparison of MMP9 enzymatic activity, by Gelatin zymography, between MCF10A, MCF7 and MDA-MB-231 cells demonstrated MCF10A and MCF7 possess little or no activity, whereas MDA-MB-231 cells have a high activity levels [76]. This potentially reflects a lower amount of MMP9 protein being produced in MCF10A and MCF7 compared to MDA-MB-231 cells. Importantly, the MMP9 activity from these cell lines directly correlates with the amount of PRMT7 expressed within them and provides additional support for the PRMT7 regulation of MMP9 expression. Furthermore, in a gene expression analysis performed in NIH 3T3 cells in which PRMT7 was knocked down, MMP9 expression was downregulated 2.3-fold [42], although this result was not validated further. It does, however, suggest that the regulation of MMP9 expression by PRMT7 may be consistent across cells and species. Importantly, we have shown that ectopic expression of MMP9 in PRMT7 depleted cells enhanced their invasive ability. This demonstrates that the reduction in cell invasion resulting from PRMT7 depletion is due, in part, to the repression of MMP9 expression. The rescue observed here was only partial, suggesting that PRMT7 may also affect breast cancer cell invasion through additional mechanisms/ pathways.

The precise mechanism through which PRMT7 is acting to regulate MMP9 expression within breast cancer cells requires further elucidation. However, there is evidence supporting both direct and indirect mechanisms through which PRMT7 may be acting. PRMT7 can influence gene expression through the methylation of histone proteins, as it has been shown to be involved in the methylation of N-terminal tail regions of histones H2A, H2B, H3 and H4 [77]. Specifically, PRMT7 affects the methylation of histone H2AR3, H2BR29, 30 and 31, H3R2 and H4R3, 17 and 19 [8, 42, 78, 79]. Importantly, MMP9 expression has been shown to be influenced by arginine methylation of histone proteins. Methylation of histone H3R17 and 26, as well as H4R3 were found to be methyl marks involved in MMP9 gene activation [80]. Therefore, PRMT7 may play a direct role in the regulation of MMP9 expression through its involvement in the methylation of histone H4R3. Alternatively, as previously described, PRMT7 was shown to negatively regulate E-cadherin expression and E-cadherin itself has been shown to affect MMP9 expression. Overexpression of E-cadherin in E-cadherin-null invasive lung epithelial tumour cell lines resulted in a decrease in MMP9 expression and secreted protein levels [81]. This also caused a reduction in their invasiveness. Conversely, loss of expression of E-cadherin in MCF10A cells resulted in a significant increase in MMP9 expression [82]. Therefore, PRMT7 potentially induces MMP9 expression indirectly through its repressive effect on E-cadherin expression. Interestingly, there appears to be an additional relationship between E-cadherin and MMP9. MMP9 has been shown to be involved in the cleavage of E-cadherin generating a soluble extracellular E-cadherin fragment (sE-cadherin: 80kDa) [83, 84]. This sE-cadherin fragment has been shown to not only have oncogenic activities itself [85, 86], but it is also capable of upregulating MMP9 expression [81, 87]. Thus creating a potential positive feedback loop that could contribute to rapidly increasing the aggressiveness of tumour cells. Furthermore, the PRMT7 protein substrate repertoire is limited and identification of novel substrates may uncover additional roles PRMT7 may play in the regulation of protein expression. Nevertheless our results have demonstrated that directly targeting and decreasing expression of PRMT7 causes reduced MMP9 expression, which specifically in breast cancer, is a therapeutically advantageous effect.

There is also evidence supporting a role for PRMT7 in an additional hallmark of cancer. PRMT7 has been shown to be involved in regulating the response of cells to cytotoxic and chemotherapeutic agents. PRMT7 was first identified in a screen for genes that confer sensitivity to cytotoxic agents, including 9-OH-E, etoposide and cisplatin [39]. Intriguingly, this study also found that reduction in PRMT7 expression caused increased sensitivity of Chinese hamster cells to UV radiation, bleomycin and camptothecin. Furthermore, in human cancer cells (HeLa) downregulation of PRMT7 expression conferred sensitivity to camptothecin treatment [40]. More recently, in NIH3T3 cells, depletion of PRMT7 was shown to cause resistance to cisplatin, mitomycin C and chlorambucin [42]. This resistance was proposed to be the results of de-repression of critical DNA repair genes (ALKBH5, APEX2, POLD1 and POLD2) following PRMT7 depletion in cells. These studies have revealed that PRMT7 is involved in regulating the cellular response to treatment with cytotoxic agents. However, the opposing findings described are highly suggestive that the cellular responses may be cell type and/or drug dependent. Further studies examining the effects of targeting PRMT7 in combination with chemotherapeutic drugs, specifically in breast and other cancer cell types, will help determine its full therapeutic value.

In summary, our results demonstrate a novel role for PRMT7 in the regulation of breast cancer cell invasion. This study established a functional role for PRMT7 in cancer, specifically in breast cancer. We have shown that PRMT7 is overexpressed in breast tumour tissues and is potentially involved in the progression and metastasis of this devastating disease. The effects observed here may have potential implications in other cancers. Identification of the molecular pathways that contribute to breast cancer development and progression has shifted the therapeutic focus to a more targeted approach [2]. Targeted therapeutic strategies offer a more refined and specific approach to the treatment of breast cancer in an attempt to reduce possible recurrence and metastasis. Our results, taken together with those of Yao et al., clearly establish PRMT7 as a key mediator of breast cancer cell invasiveness and demonstrate that PRMT7 represents a novel biomarker and may be a promising new therapeutic target.

## MATERIALS AND METHODS

### Cell lines

Breast cancer cell lines were from the American Type Culture Collection (Manassas, VA). BT20, BT549, MDA-MB-231 and 293T cells were cultured Dulbecco's Modified Eagle's Medium (DMEM) with 2 mM glutamine, 1mM sodium pyruvate and 10% fetal bovine serum (FBS). MCF7 and Hs578T media were also supplemented with 2.75 μg/mL insulin. MCF10A cells were obtained from Drs. Marie-Chloé Boulanger and Josee Lavoie (Laval University Cancer Research Center, Laval, QC) and were cultured as in [88].

### Antibodies and Reagents

Human polyclonal PRMT7 antibodies were from Imgenex or Upstate Inc. α-tubulin antibody was purchased from Sigma-Aldrich. GAPDH antibody was purchased from Covance.

### Immunohistochemistry

Tissue microarray slides were purchased from US Biomax, Inc. (Rockville, MD, USA). For PRMT7 staining, antigen retrieval was performed using sodium citrate (pH 6) and microwave treatment. Primary PRMT7 rabbit polyclonal antibody from Imgenex (Cat. # IMG-5121A, Lot# 02081856A) was used at a dilution of 1:50 and was detected with the Envision Polymer Detection System (Dako, Mississauga, ON, Canada). A scoring system from 0 to 3 based on the intensity of PRMT7 cytoplasmic staining in normal breast epithelial cells and epithelial-derived breast tumor cells was used, with 3 being the strongest staining. Distribution of the staining in the tissue was scored from 0 to 3; 0 (None), 1 (<30%), 2 (30-70%), 3 (>70%). These scores were multiplied to determine the composite score used in the final assessment. Composite scores ranging from 0-2 represent low expression, 3-5 represented moderate expression and scores 6 or greater (6+) represented high expression. Scoring was performed independently by two pathologist.

### DNA Constructs

PRMT7-mGFP and PRMT7-MycDDK in the pLenti-C vector, shControl, shPRMT7-1 and shPRMT7-2 in the pGIPZ vector, and the pCMV-XL5-MMP9 were purchased from Origene Inc. Luciferase was introduced stably into cells using a lentiviral construct that drives constitutive expression (pLenti PGK V5-LUC, Addgene).

### Lentivirus production

293T cells were transiently transfected with packaging plasmids, pMD2.G and pPAX2 along with the expression plasmid. After 24 h, media on these cells was changed to fresh media and target cells were plated (250 000 cells in a 10 cm plate). Following an additional 24 h incubation, lentivirus was harvested from the media by filtering it through a 0.45 μm syringe filter. Media containing virus with 8 μg/mL polybrene was placed on target cells and allowed to infect for 48 h. Media was then changed and cells were selected (puromycin) until a control plate containing uninfected cells were fully selected (48-72 h). Stock cells were maintained under selection.

### MTT assays

MTT assays were carried out as described in [89]. Absorbances were acquired using a Spectramax M2 microplate reader with Softmax Pro software.

### Cell counts

Cells were initially seeded at equal numbers and viable cell numbers determined at days 1, 4 and 8.

### Western blot analysis

Western blotting was performed similar to those described [90]. Band densities were determined using images that were within the linear range of signal intensity.

### Cell motility and invasion assays

For Transwell chamber assays, 24 well BD Control chambers or Biocoat Matrigel invasion chambers (BD Biosciences, Mississauga, ON, Canada) were used according to the manufacturer's protocol. To measure cell motility, cells were seeded into BD Control chambers which contain a membrane with 8 μm pores at the bottom allowing for cells to migrate through to the underside of the chamber. To assess cell invasion, cells were seeded into BD Biocoat Matrigel invasion chambers. These chambers differ from the Control chambers in that a Matrigel layer is applied to on top of the membrane, therefore providing a physical barrier that cells are required to penetrate in order to reach the pores and the underside of the chamber membrane. Fifty thousand cells in serum-free DMEM were seeded into each chamber and placed into a well of a 24 well plate containing 500 μL complete DMEM. Cells were then incubated for 24 h (MDA-MB-231 cells) or 72 h (MCF7 cells). Following incubation, cells on the upper surface of the membrane were removed by scrubbing within the chamber. Cells that reached the underside of the chamber membrane were fixed and stained using the Diff-Quik staining kit (Allegiance Inc.). A minimum of six random fields, at 40X magnification, were counted to determine cell numbers.

### Immunofluorescence and fluorescence imaging

Cells were seeded onto glass coverslips in a 6 well plate and incubated overnight allowing them to adhere. Cells were washed twice with 1XPBS (phosphate buffered saline, PBS) before fixation in 4% paraformaldehyde in PBS (10 min) at room temperature. Cells were then washed three times with PBS and subsequently permeabilized for 10 min at room temperature using 0.5% Triton-X-100 in PBS. Cells were then washed three times using PBS and blocked for one hour with 5% milk in PBS. Cells were then washed once with PBS and incubated with primary antibody (1:50 dilution) at room temperature for one hour. Following the incubation, cells were washed once with 0.1% Triton-X-100 in PBS and twice more with PBS. Cells were then incubated with secondary antibody (goat anti-rabbit Alexa 594) for 1 hour at room temperature. Washes were performed again with 0.1% Triton-X-100 in PBS and twice with PBS. Coverslips were mounted onto glass slides using Vectashield Mounting Media with DAPI (Vector Laboratories Inc.). Cells expressing PRMT7-GFP fusion protein were fixed with 4% paraformaldehyde in PBS for 15 minutes, permeabilized with 0.5% Triton X-100 in PBS for 10 minutes and washed 3 times with PBS and cover slips were mounted onto glass slides using the Vectashield Mounting medium with DAPI (Vector Laboratories).

### Mircroscopy

Phase images for invasion assays were acquired using a Zeiss Axiovert 40 CFL inverted microscope using a Canon digital camera. Fluorescence imaging was performed on a Ziess Axio Imager. Z1 microscope and images acquired with an AxioCam HRm camera driven by Zeiss Axiovision 4.5 software.

### *In vivo* experimental metastasis model and *in vivo* imaging system

All the animal experiments were approved by and carried out according to the University of Ottawa Animal Care Committee and Animal Care and Veterinary Services. Six week old female NOD.CB17-Prkdc^scid^/NrcCrl SCID mice (Charles River, Montreal, QC, Canada) were injected intravenously into the tail with MDA-MB-231 cells stably expressing a non-targeting control shRNA and Luciferase or a PRMT7-targeted shRNA and Luciferase. Four mice per group were randomly assigned. Five hundred thousand cells in 100 μL of PBS were injected into the tail vein of each mouse. For bioluminescence imaging, mice were given a dose of D-luciferin (150 mg/kg) in PBS by intraperitoneal injection and then anesthetised with isoflurane. At 10 min after injection, bioluminescence was detected with an *in vivo* imaging system (IVIS Spectrum, Caliper Life Sciences by PerkinElmer, Waltham, MA, USA). Images were acquired and quantitated with Living Image 4.3.1 software. After final imaging, mice were euthanized and were dissected for lung staining and extraction. India Ink stain (15% India Ink, 85% water, 3 drops of NH_4_OH/100mL) was injected in through the trachea and down into the lungs. This resulted in black staining of the normal lung tissue while the tumour cell nodules were impenetrable to the stain and remain white in color. Lungs are then extracted, washed in water for 5 min and then incubated once for 5 min in Fekets solution (300 mL 70% ethanol, 30 mL 37% formaldehyde, 5 mL glacial acetic acid, water to 500 mL total), followed by an overnight incubation in fresh Fekets solution at room temperature to fix the tissue. Lungs were then imaged using a canon digital camera.

### Reverse transcriptase (RT) PCR analysis

Total RNA was isolated from cells using TRIzol (Life Technologies, Burlington, ON, Canada). First strand cDNA synthesis was performed using AMV Reverse Transcriptase (Promega, Madison, WI, USA). PCRs were performed in a 15 μL reaction using GoTaq Green Master Mix (Promega). For a list of primers used in this study see Supplementary Table 1. Band densities were determined using images that were within the linear range of signal intensity and normalized to the endogenous reference gene (GAPDH). Values were then expressed as changes in expression relative to the experimental control.

### Quantitative RT PCR analysis

Total RNA was extracted from cells and cDNA was generated as described above. Quantitative RT-PCR was performed in a Life Technologies Applied Biosystems (ABI) 7500 Real-time PCR system (Burlington, ON, Canada) using FastStart SYBR Green master mix (Roche, Basel, Switzerland). PCRs were performed in 96 well plate format in triplicate. Data acquisition and analyses were performed using ABI 7500 software v2.0.6. Primer pairs used for quantitative RT-PCR are indicated in Supplementary Table 1. GAPDH was used as the endogenous reference gene.

### MMP array

Cells (500 000) were plated into 6 well plates in complete media. After 24 h, cells were washed and incubated in 1mL of serum free media for 48 h. Cell-conditioned media was collected and placed on membrane based MMP arrays to assess secreted protein levels (Ray Biotech, Inc, Norcross, GA, USA, Cat. #AAH-MMP). Arrays were used according to manufacturer's specifications. Briefly, conditioned media was placed on a membrane upon which MMP antibodies were present and incubated overnight at 4°C. Following this incubation, membranes were washed and incubated with a biotinylated antibody cocktail. The membranes were once again washed and then incubated with HRP-sterptavidin. Following a final wash procedure, MMP proteins levels were visualized by chemiluminescence.

### Gelatin Zymography

Cell-conditioned medium was analyzed for MMP9 activity by Gelatin zymography. Cells were incubated in serum-free media for 24 h and then the conditioned medium was collected and concentrated. Samples were prepared in non-reducing conditions in 5× sample buffer (0.625 M Tris-HCl, 10% glycerol, 2% SDS, 2% Bromphenol Blue) and resolved on 8% SDS-PAGE gels containing 0.5 mg/ml of gelatin Type A from porcine skin (Sigma-Aldrich). The gels were then washed twice in 2.5% (v/v) Triton X-100 for 15 min each at room temperature. Following this, gels were incubated in developing buffer (100 mM Tris-HCl, pH 7.9, 30 mM CaCl_2_, and 0.02% sodium azide) for 30 min at room temperature followed by a second incubation in fresh developing buffer overnight at 37°C. The gels were then stained with SimplyBlue SafeStain solution (Life Technologies) for 15 min, followed by de-staining in water to reveal areas of activity [91].

### Statistical analysis

Statistical analysis was performed using the Student's *t* test, where *p* < 0.05 was considered statistically significant. Immunohistochemical data was evaluated for significance using both Chi squared (χ^2^) test and ANOVA, *p* < 0.05 was considered statistically significant.

## SUPPLEMENTARY MATERIAL FIGURES AND TABLE


