# Supplementary material for: Protein arginine methyltransferase 7 promotes breast cancer cell invasion through the induction of MMP9 expression
Source: Oncotarget. 2014 Dec 26;6(5):3013–32. doi: 10.18632/oncotarget.3072 (PMC4413634; doi:10.18632/oncotarget.3072)
Supplement: Supplementary file 1 [file oncotarget-06-3013-s001.pdf]

Protein arginine methyltransferase 7 promotes breast cancer cell invasion through the induction of MMP9 expression

Supplementary Material

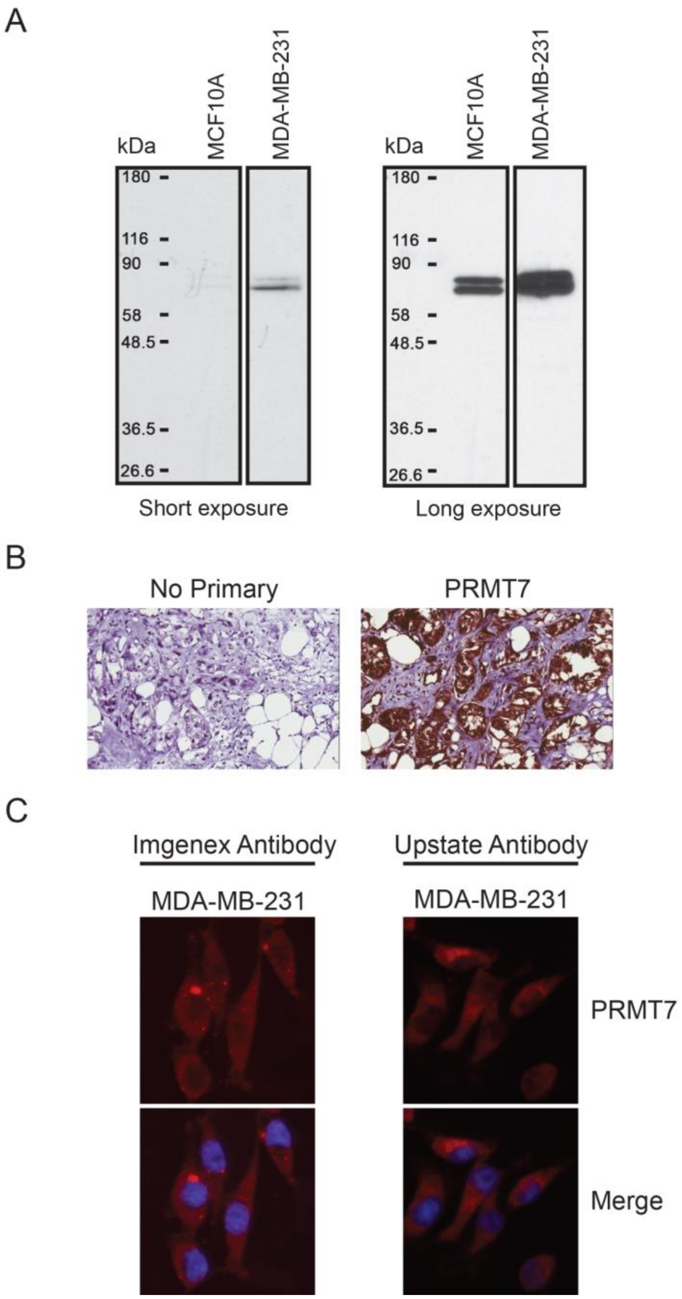

Supplementary Figure 1: Validation of PRMT7 antibody activity and specificity used in immunohistochemical analysis. Western blot analysis using the Imgenex Inc. PRMT7 rabbit

polyclonal antibody shows specific detection of bands corresponding to PRMT7 (A). Images of representative short and long exposures are shown. B, shows immunohistochemical staining of primary breast tumour tissue with no primary antibody as a control or using the PRMT7 rabbit polyclonal antibody. This demonstrates that the staining observed is specific to the presence of the PRMT7 antibody, with limited or no non-specific contribution from the secondary antibody. Immunofluorescence for endogenous PRMT7 in MDA-MB-231 cells (C). This was done using two different commercially available PRMT7 antibodies, the rabbit polyclonal antibody from Imgenex Inc. (Left), and the rabbit polyclonal antibody from Upstate Inc. (Right).

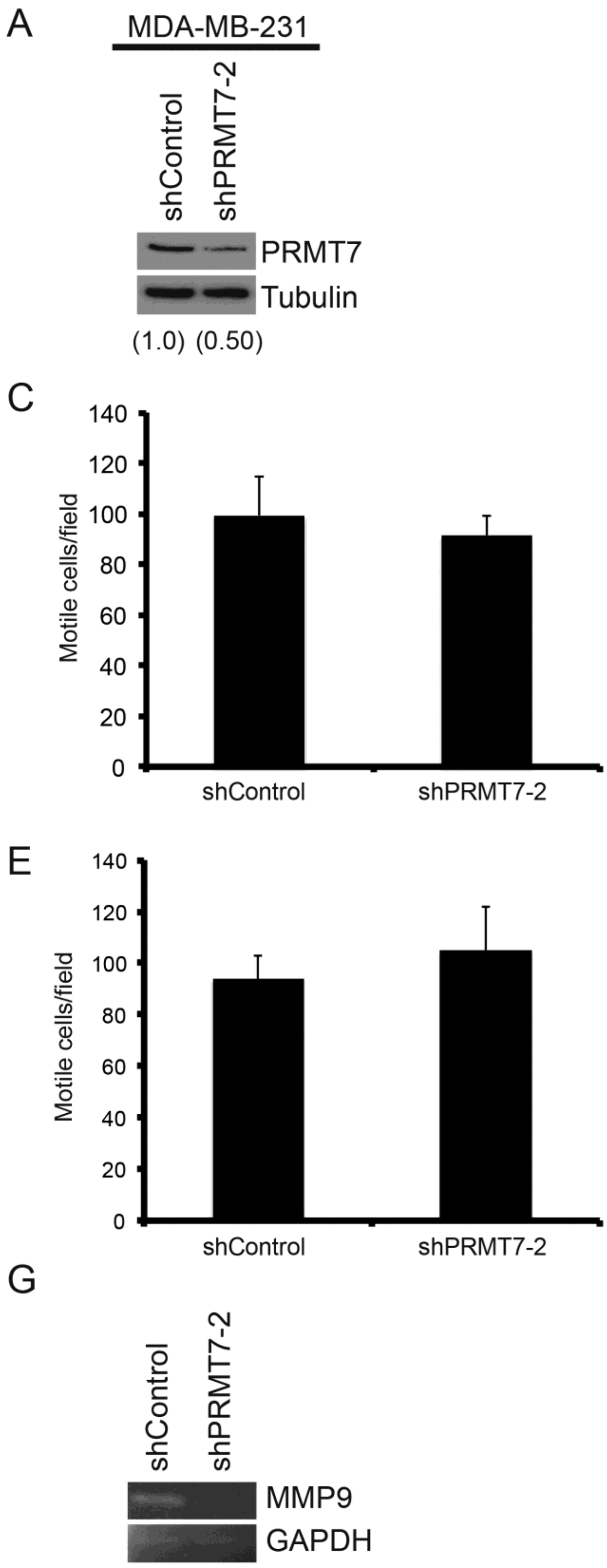
A
MDA-MB-231
shControl
shPRMT7-2
PRMT7
Tubulin
(1.0) (0.50)
B
BT549
shControl
shPRMT7-2
PRMT7
Tubulin
(1.0) (0.43)
C
Motile cells/field
shControl
shPRMT7-2
D
Invasive cells/field
**
shControl
shPRMT7-2
E
Motile cells/field
shControl
shPRMT7-2
F
Invasive cells/field
*
shControl
shPRMT7-2
G
shControl
shPRMT7-2
MMP9
GAPDH

Supplementary Figure 2: Assessment of motility and invasion with a second shRNA targeting PRMT7. Stable depletion of PRMT7 from MDA-MB-231 and BT549 cells using a second unrelated lentiviral introduced shRNA (shPRMT7-2) efficiently reduces protein levels (A and B). Densitometry of the band intensities are indicated below in parentheses. Cell motility and invasion were assessed as previously described in Figure 3. Depletion of PRMT7 using shPRMT7-2 did not significantly affect the motility of invasive breast cancer cells, MDA-MB-231 (C) and BT549 (E). A significant reduction in their ability to invade was observed (MDA-MB-231 (D) and BT549 (F)). Data represents the mean ± standard error of five independent experiments for MDA-MB-231 cells and four independent experiments for BT549 (*$p < 0.05$, **$p < 0.01$ comparing to control). Total RNA was isolated from MDA-MB-231 cells stably depleted of PRMT7 using shPRMT7-2 and assessed for mRNA levels by PCR analysis of MMP9 expression (G). GAPDH serves as a loading control.

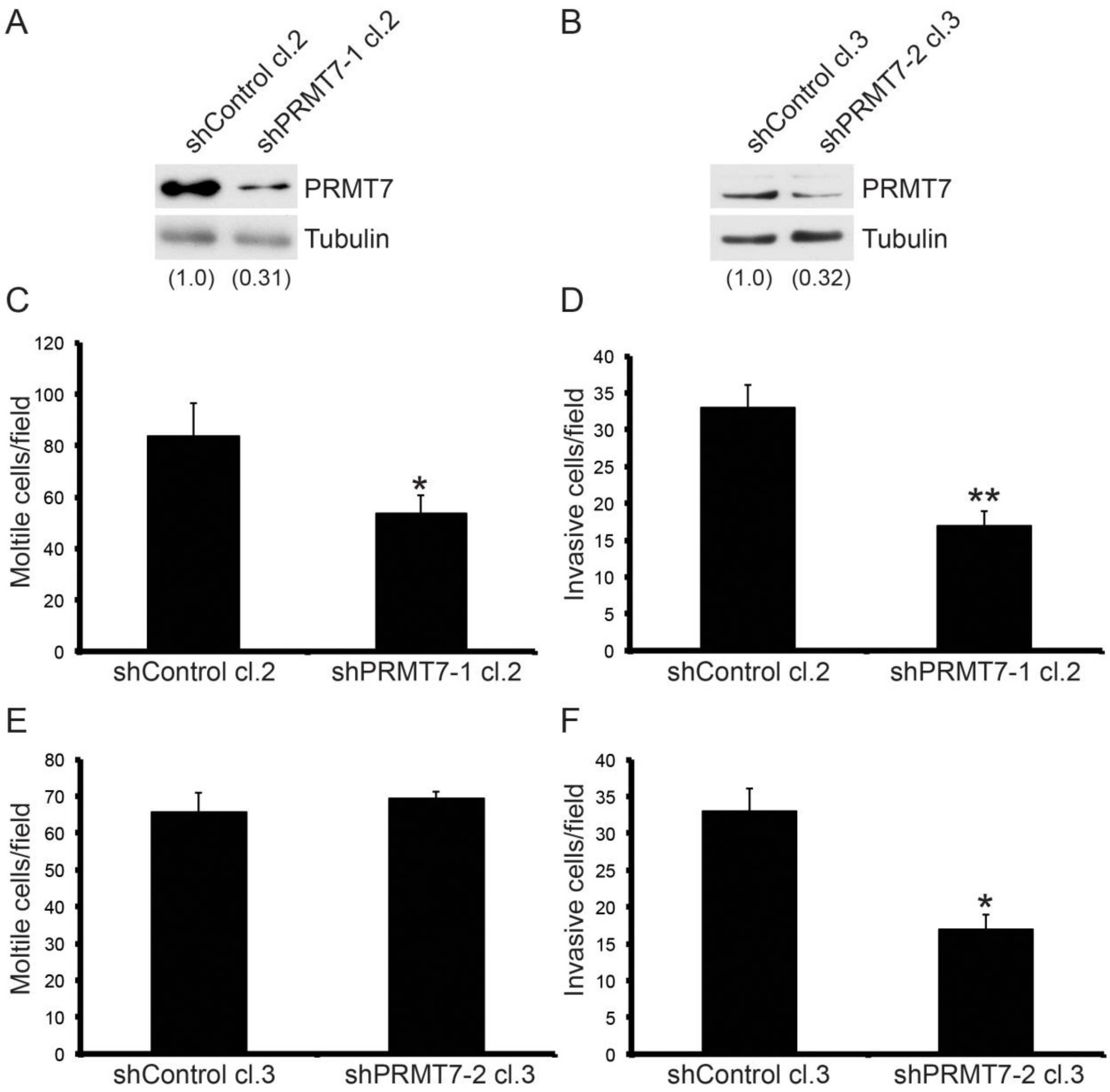

Supplementary Figure 3: Assessment of motility and invasion in clonal populations of MDA-MB-231 cells stably depleted of PRMT7. Stable clones of MDA-MB-231 cells expressing non-targeting shRNA (shControl) or one of two unrelated PRMT7 targeting shRNAs (shPRMT7-1 and shPRMT7-2) were also generated by lentiviral infection and colonies were selected. Selected clonal populations show efficient PRMT7 protein depletion with shPRMT7-1 (cl. 2, A)

and shPRMT7-2 (cl. 3, B). Densitometry of the band intensities are indicated below in parentheses. Cell motility and invasion was assessed as previously described in Figure 3. Consistent with the PRMT7 depleted cell populations, clonal populations depleted of PRMT7 had a significant reduction in their ability to invade (shPRMT7-1 cl. 2 (D) and shPRMT7-2 cl. 3 (F)), with minimal effects on motility (C and E). Data represents the mean ± standard error of five independent experiments for shPRMT7-1 cl. 2 cells and four independent experiments for shPRMT7-2 cl. 3 cells (* $p < 0.05$, ** $p < 0.01$ comparing to control).

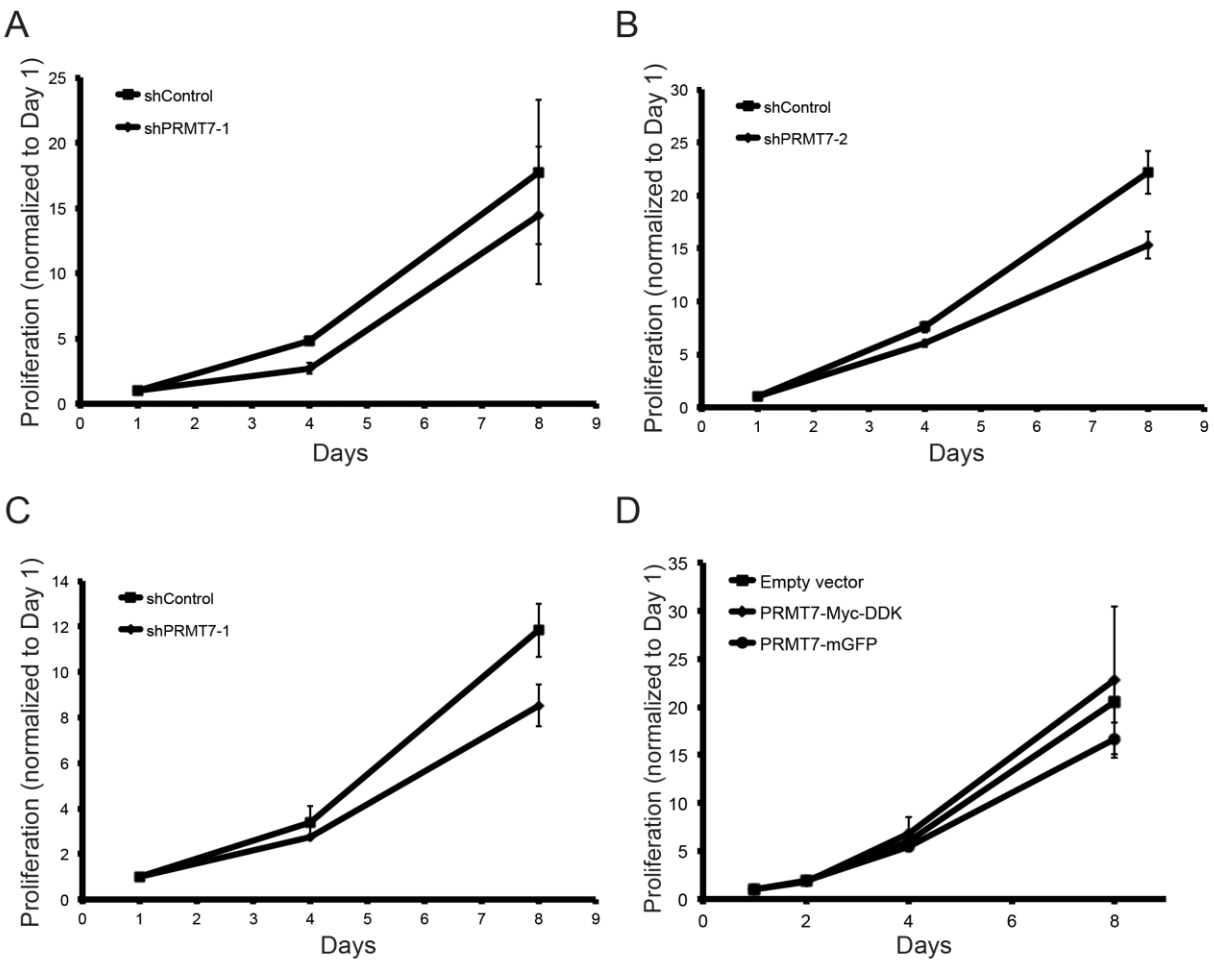

Supplementary Figure 4: Effect of PRMT7 on breast cancer cell proliferation. Cell proliferation was determined in MDA-MB-231 (A and B) and BT549 (C) cells depleted of PRMT7 by viable cell counts. Cells expressing a non-targeting shRNA were used as controls. Cells were initially plated at equal numbers and counted at days 1, 4 and 8. Proliferation of MCF7 cells overexpressing PRMT7-MycDDK or PRMT7-mGFP was measured by MTT assay (D). Initially cells were plated at equal numbers and lysed and analyzed on days 1, 2, 4 and 8 post plating. Cells expressing an empty vector were used as controls. Data is the mean ± standard error of three independent experiments.

Supplementary Table 1: PCR Primer List

Gene	Product size	Forward sequence	Reverse sequence	RT-PCR	Q-RT-PCR
GAPDH	452 bp	ACCACAGTCCATGCCATCAC	TCCACCACCCTGTTGCTGTA	x	x
MMP9	179 bp	TTGACAGCGACAAGAAGTGG	GCCATTCACGTCGTCCTTAT	x	
MMP9	881 bp	TCTATGGTCCTCGCCCTGAA	TTGTATCCGGCAAACTGGCT	x	
MMP9	158 bp	GCCTCTGGAGGTTCGACG	ACTCACGCGCCAGTAGAAG	x	x
PRMT7	655 bp	TGAACATGGGCAGCACATCGC	GCAAGGTCATCTTATGCAGAT	x	
